# Effects of irrigation and nitrogen on chlorophyll content, dry matter and nitrogen accumulation in sugar beet (*Beta vulgaris* L.)

**DOI:** 10.1038/s41598-021-95792-z

**Published:** 2021-08-17

**Authors:** Ning Wang, Fengzhen Fu, Hongrong Wang, Peng Wang, Shuping He, Hongying Shao, Zhen Ni, Xingmei Zhang

**Affiliations:** 1 Postdoctoral Research Station of Crop Science, Heilongjiang Bayi Agricultural University, Daqing, 163319 People’s Republic of China; 2 College of Horticulture and Landscape, Heilongjiang Bayi Agricultural University, Daqing, 163319 People’s Republic of China; 3 Agriculture College, Heilongjiang Bayi Agricultural University, Daqing, 163319 People’s Republic of China

**Keywords:** Physiology, Plant sciences

## Abstract

A 2-year field experiment was conducted to analyze the growth conditions, physical features, yield, and nitrogen use efficiency (NUE) of sugar-beet under limited irrigation conditions in northeast of China. A cultivar H003 was used as plant materials; six treatments (C1–C6) were included: C1, no nitrogen applied, rain-fed; C2, nitrogen (120.00 kg ha^−1^), rain-fed; C3, no nitrogen applied, hole irrigation for seeding; C4, nitrogen (120.00 kg ha^−1^), hole irrigation for seeding; C5, no nitrogen applied, hole irrigation for seeding; and C6, nitrogen (120.00 kg ha^−1^), hole irrigation for seeding, and irrigation at foliage rapid growth stage. The irrigation supply was only 500 mL/plant once. Results showed C6 showed the highest chlorophyll content, dry matter accumulation, yield, etc. and had the best NUE among all the treatments. In conclusion, under the routine fertilization conditions of northeast of China, the cultivation measure of hole irrigation 500 mL/plant for seeding combined with irrigation 500 mL/plant at foliage rapid growth stage greatly improved sugar-beet yield and NUE.

## Introduction

Russia, Ukraine, France and the United States are among the top countries in the world in terms of sugar beet production^1–3^, and China ranks the seventh^4–6^. Sugar beet is an important cash crop in the northwest, northeast and north China^7–9^. A previous study^10^ suggests that sugar beet is one of highly water-hungry crops, and each plant is capable of releasing 1 L of water a day into the atmosphere at the foliage rapid growth stage. So, adequate water is necessary for the growth of sugar crops^11–15^. Sugar-beet is also a fertilizer-loving crop^16–19^. Among all the nutrients, nitrogen is the most important one that affects sugar yield, while excessive or deficient nitrogen will greatly decrease the output of sugar beet^20–22^. Too much nitrogen application always improves the vegetative growth of sugar beet, which inhibits the translocation of dry matter from vegetative organs to tuber and then reduced the final yield of sugar beet. In addition, too much nitrogen also greatly decreased the sugar content and quality of tubers^23–25^. Lin^26^ conducted a field experiment in northeast of China and found that tuber yield was positively correlated with irrigation amount, and sugar yield was positively correlated with nitrogen application rate; the relationship between tuber yield and nitrogen and the relationship between sugar yield and irrigation amount both showed linear quadratic models. These results indicate that the temperate humid monsoon climate of northeast of China is beneficial to sugar beet. Previous studies also demonstrated that the coupling of irrigation and nitrogen effectively improved the growth vigor and yield of many crops, such as melon^27,28^, angelica^29^, tomato^30–32^, winter rape^33,34^, wheat^35,36^, and corn^37–39^.

In northeast of China, most rainfalls occur in July–September every year, which cannot meet for the growth of sugar beet because sugar beet grows fast in June. Currently few researchers focus on the nitrogen deficiency and water limitation of sugar beet production in the region. Here we studied the physiological characteristics, yield, and nitrogen use efficiency (NUE) of sugar beet under the conditions of limited irrigation and (or) nitrogen supplement, aiming to better understand the effects of nitrogen and water on sugar beet in northeast of China.

## Materials and methods

### Materials

The sugar beet cultivar Dutch H003 (Syngenta, Germany) was used as plant materials in the study. The chemical fertilizers were urea (N ≥ 46%, Petro-China), tripe superphosphate (P ≥ 20%,Yuntianhua, China) and potassium sulfate (K ≥ 41%,Hongniu, German).

### Experiment design

Experiments were conducted at the experimental farm of Heilongjiang Bayi Agricultural University (46° 37′ N, 125° 11′ E) in 2016 and 2017. The soil type is Humic Calcaric Cambisols^40^ and the fertility is equal everywhere. The properties of 0–20 cm surface soil were pH 8.67 and 8.42; organic matter content 33.4 mg kg^−1^and 33.5 mg kg^−1^, available nitrogen 129.98 mg kg^−1^and 136.02 mg kg^−1^, available phosphorus 31.41 mg kg^−1^ and 30.55 mg kg^−1^, and available potassium 171.54 mg kg^−1^and 179.00 mg kg^−1^ in 2016 and 2017 respectively. The typical local fertilization amount for sugar beet was: N (120.00 kg ha^−1^); P (45.85 kg ha^−1^); and K (100.00 kg ha^−1^).

Six treatments (C1–C6) were set and the details were: C1, no nitrogen applied, rain-fed; C2, nitrogen (120.00 kg ha^−1^), rain-fed; C3, no nitrogen applied, hole irrigation for seeding; C4, nitrogen (120.00 kg ha^−1^), hole irrigation for seeding; C5, no nitrogen applied, hole irrigation for seeding, and watering in sugar beet growth period; and C6, nitrogen (120.00 kg ha^−1^), hole irrigation for seeding, and watering in sugar beet growth period. P (45.85 kg ha^−1^) and K (100.00 kg ha^−1^) was applied in the six treatments according to local fertilization levels.

Randomized block with three repeats were adopted. Each plot consisted of four rows. The row was 8 m long with row spacing of 0.65 m. Plants within a row was 0.20 m apart, and the density was 76,950 plant ha^−1^. Sugar beet was sowed on May 1 and harvested on October 11 in 2016 and sowed on May 5 and harvested on October 14 in 2017. In both year, ridge culture combined with direct-seeding was employed, and all the fertilizers were used as basal fertilizer and applied at 5 cm away from ridges and 15 cm soil depth. The amount for hole irrigation for seeding and supplemental irrigation during growth period was 500 mL/plant. And the supplemental irrigation for C5 and C6 treatments was conducted at six-leaf stage (foliage rapid growth stage) of sugar beet and watered in a hole of 12 cm depth and 5 cm of diameter.

### Measure parameters and methods

The soil plant analysis development (*SPAD*), tuber sugar content, dry matter accumulation, nitrogen content of sugar beets were all measured at tuber growth, sugar accumulation and harvest stages. The *SPAD* value was measured by a chlorophyll analyzer (*TYS-A*, Zhejiang Top Instrument Co., Ltd., China), three plants per plot; the sugar content was determined by a digital refractometer (*PAL-1*, ATAGO, Japan), three root tubers per plot. Three well-developed plants were sampled from each plot for dry matter measurement. After washed, the leaves and tuber roots were separated, subjected to 105 ℃ for 30 min for enzyme inactivation and dried to constant weight at 75 ℃. Kjeldahl method was used to determinate the nitrogen content in plants by an automatic Kjeldahl Apparatus (*KJELTEC 2300*, FOSS, Sweden).

Finally, the nitrogen use efficiency was calculated according to the following equation:

$$\begin{aligned} & {\text{Nitrogen}}\;{\text{use}}\;{\text{efficiency}}\;\left( \% \right) = \left( {\left( {{\text{Total}}\;{\text{nitrogen}}\;{\text{amount}}\;{\text{of}}\;{\text{plants}}\;{\text{in}}\;{\text{nitrogen}}\;{\text{treatment}}\;\left( {{\text{kg}}\;{\text{ha}}^{{ - {1}}} } \right)} \right.} \right. \\ & \quad \left. { - \,{\text{Total}}\;{\text{nitrogen}}\;{\text{amount}}\;{\text{of}}\;{\text{plants}}\;{\text{in}}\;{\text{treatment}}\;{\text{without}}\;{\text{nitrogen}}\;\left( {{\text{kg}}\,{\text{ha}}^{{ - {1}}} } \right)} \right)/{\text{Nitrogen}}\;{\text{applied}}\;{\text{amount}}\;\left( {{\text{kg}}\,{\text{ha}}^{{ - {1}}} } \right)*{1}00\% . \\ \end{aligned}$$

### Data analysis

**Figure 1:**
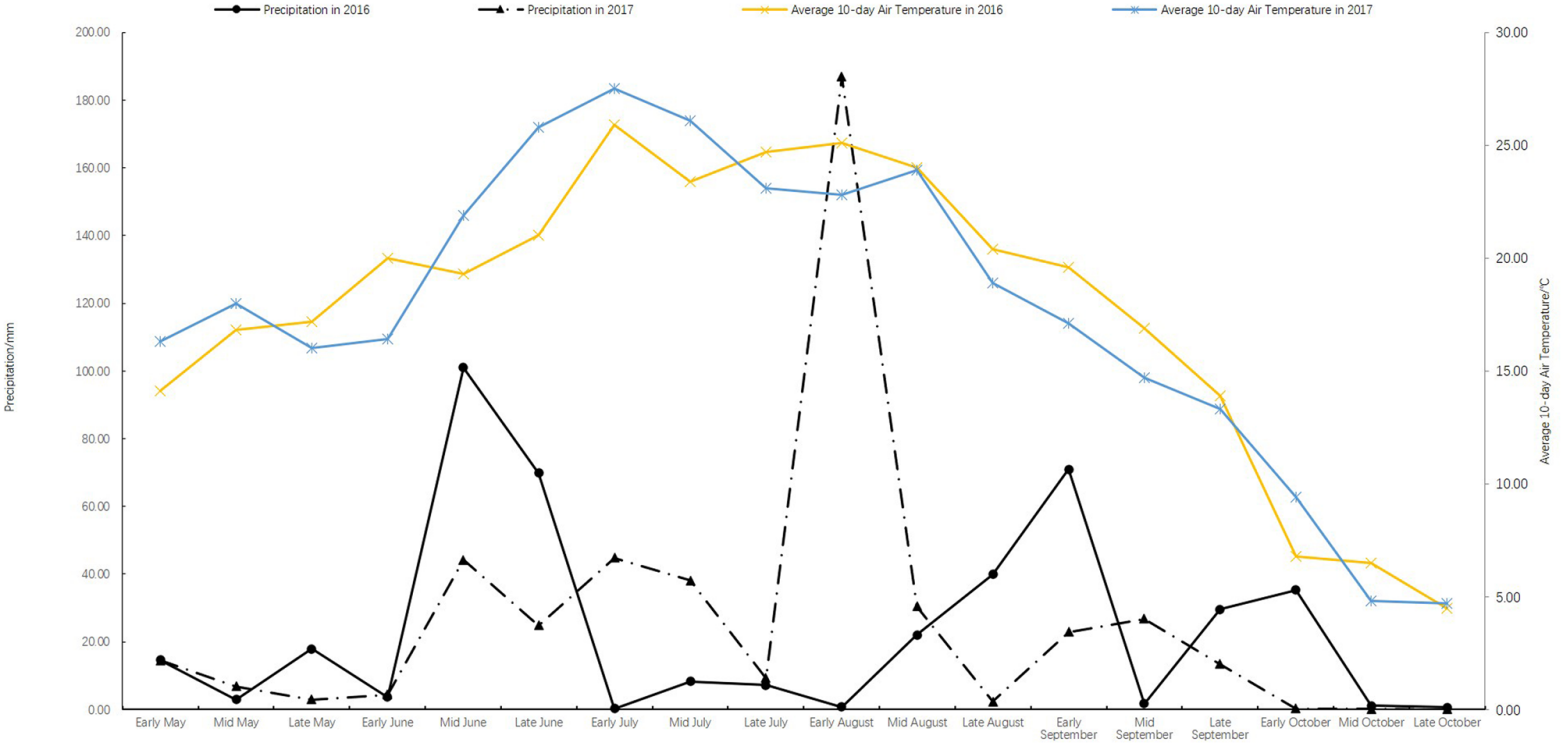
Precipitation and average 10-day temperature in sugar beet growth period in 2016 and 2017.

Data were analyzed using SPSS 21.0 (*IBM*, USA) and Excel 2016 (*Microsoft*, USA), and *T-* test was used to determine the significance of difference. Rainfall and average 10-day temperature in sugar beet growth period (from May to October) are presented in Fig. 1. These data were obtained from a small meteorological station at the experimental farm. The total active accumulated temperature, rainfall, and evaporation capacity from May to October was 2815.3 ℃, 427.8 mm and 581.2 mm respectively in 2016 and was 2872.7 ℃, 473.0 mm and 634.4 mm respectively in 2017.

### Ethical statements

Experimental research and field studies on sugar beet plants, including the collection of plant material, comply with the IUCN Policy Statement on Research Involving Species at Risk of Extinction and the Convention on the Trade in Endangered Species of Wild Fauna and Flora.

## Results

### Effect of limited irrigation on chlorophyll content of sugar beet

**Figure 2:**
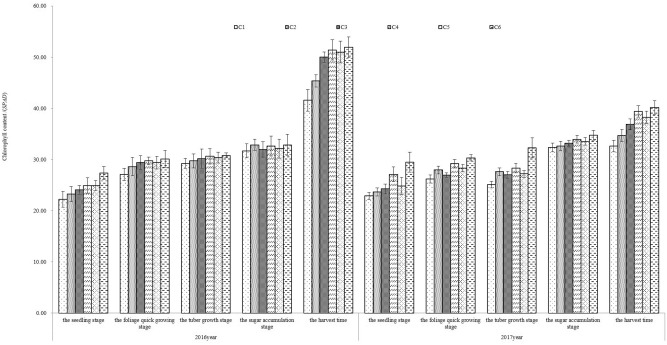
Effect of limited irrigation on *SPAD* of sugar beet leaves in 2016 and 2017.

The chlorophyll content of sugar beet is closely related to cultivar characteristics, soil moisture and fertilization^41^. The *SPAD* of leaves is presented in Fig. 2. In all treatments, the leaf chlorophyll content increased with sugar beet growing and reached the maximum at harvest stage. At every growth stage, the leaf chlorophyll content of all treatments was ranked as: C6 > C4 > C5 > C3 > C2 > C1, which indicated that both nitrogen amount and irrigation improved the chlorophyll content of sugar beet. At seedling stage, the leaf chlorophyll content in the treatments employing hole irrigation for seedling measure (C3–C6) was 6.66–14.98% and 12.66–19.76% more than that in rain-fed treatments (C1 and C2) in 2016 and 2017, respectively. There was no chlorophyll difference between C1 and C2 and between C3, C4, C5 and C6 (*p* < 0.05). The result indicated that the cultivation method of hole irrigation for seeding helped to increase leaf chlorophyll content. The plants in treatments with nitrogen supplied (C2, C4 and C6) were more vigor and had more leaf chlorophyll content than in no nitrogen application treatments (C1, C3, and C5), and the maximum chlorophyll contents in nitrogen supplied treatments were 1.79–8.38% and 4.83–6.41% more than those in treatments without nitrogen applied in 2016 and 2017, respectively. The 2-year experiment showed that when the nitrogen was at the same level, more water supply would increase the chlorophyll content more, and the chlorophyll content of all treatments showed the trend of hole irrigation for seedling + irrigation > hole irrigation for seeding > rain-fed.

### Limited irrigation improving dry matter accumulation in sugar beet

#### Dry matter accumulation in roots

**Figure 3:**
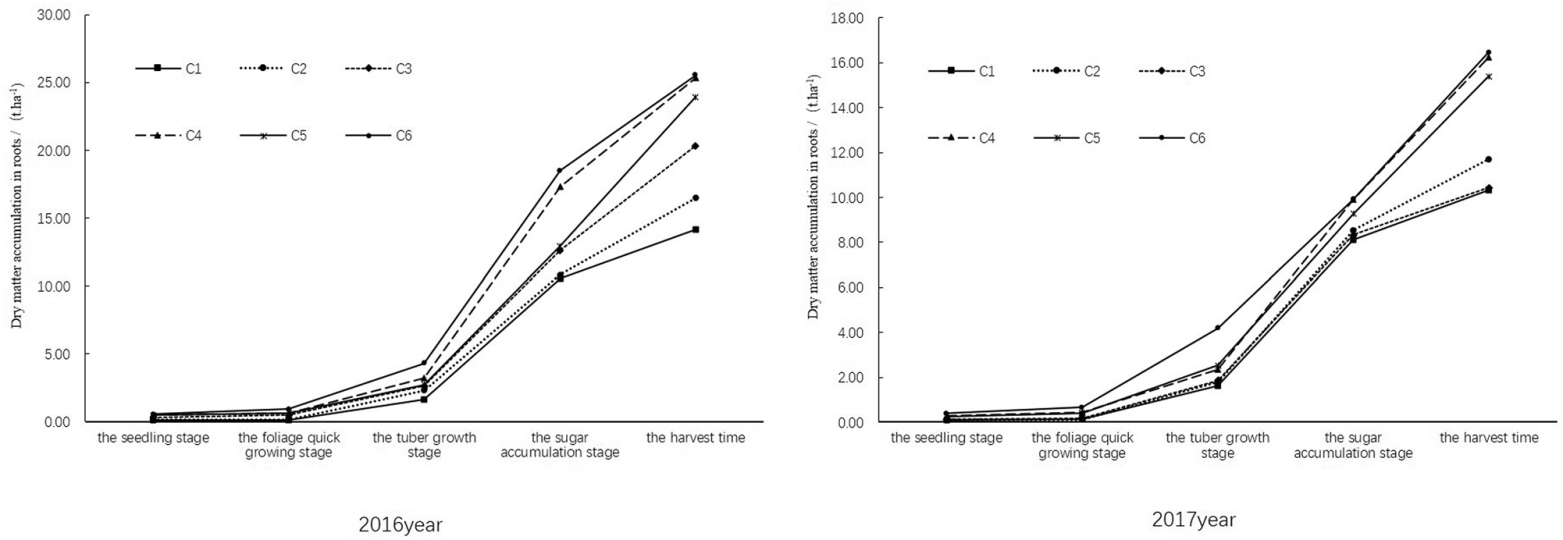
Effect of limited irrigation on dry matter accumulation in the roots of sugar beet in 2016 and 2017.

In both years, dry matter accumulation in tubers increased with sugar beet growing. The dry matter accumulation grew slowly from seeding to foliage rapid growth stage while rapidly increased at later growth stages (Fig. 3). At seedling stage, the dry matter accumulation in tubers of C1 and C2 was significantly less than that of other treatments (C3–C6) (*p* < 0.05). But no dry matter difference was observed between C1 and C2 (*p* > 0.05) and between C3–C6 (*p* > 0.05). At foliage rapid growth stage, the dry matter accumulation in tubers was significantly different between treatments (*p* < 0.05) and showed the trend of hole irrigation for seedling + irrigation > hole irrigation for seeding > rain-fed. Nitrogen application treatments (C2, C4 and C6) showed higher dry matter accumulation in tubers than no nitrogen treatments (C1, C3, and C5).

At tuber growth and sugar accumulation stages, the dry matter accumulation in tubers from high to low was in the order of C6 > C5 > C4 > C3 > C2 > C1. At mature stage, the sugar beet of C6 accumulated 25.55 ± 1.43 t ha^−1^ and 16.45 ± 0.41 t ha^−1^ dry matter in tubers in 2016 and 2017, respectively. The result indicated that hole irrigation for seedling and irrigation at later growth stages were both conductive to dry matter accumulation in tubers. For the three treatments with nitrogen applied (C2, C4 and C6), the dry matter accumulation in tubers of C6 was 0.74% and 1.28% more than that of C2 in 2016 and 2017 respectively, and was 35.54% and 28.81% more than that of C4 in 2016 and 2017 respectively. For no nitrogen application treatments (C1, C3 and C5), C5 showed 14.97% and 32.27% more dry matter accumulation in tubers than C1 in 2016 and 2017 respectively, and achieved 40.76% and 32.98% more dry matter in tubers than C3 in 2016 and 2017 respectively. As for the effect of nitrogen application, C6 (nitrogen application) achieved 6.38% more dry matter accumulation in tubers than C5 (no nitrogen) in both years; C4 got 38.78% and 28.19% more dry matter accumulation in tubers than C3 in 2016 and 2017, respectively; compared with C1, nitrogen supplement in C2 increased dry matter accumulation in tubers by 13.96% in 2016 and by 11.87% in 2017. These results indicated that both water supplement and nitrogen application improved the dry matter accumulation in sugar beet tubers.

#### Dry matter accumulation in leaves

**Figure 4:**
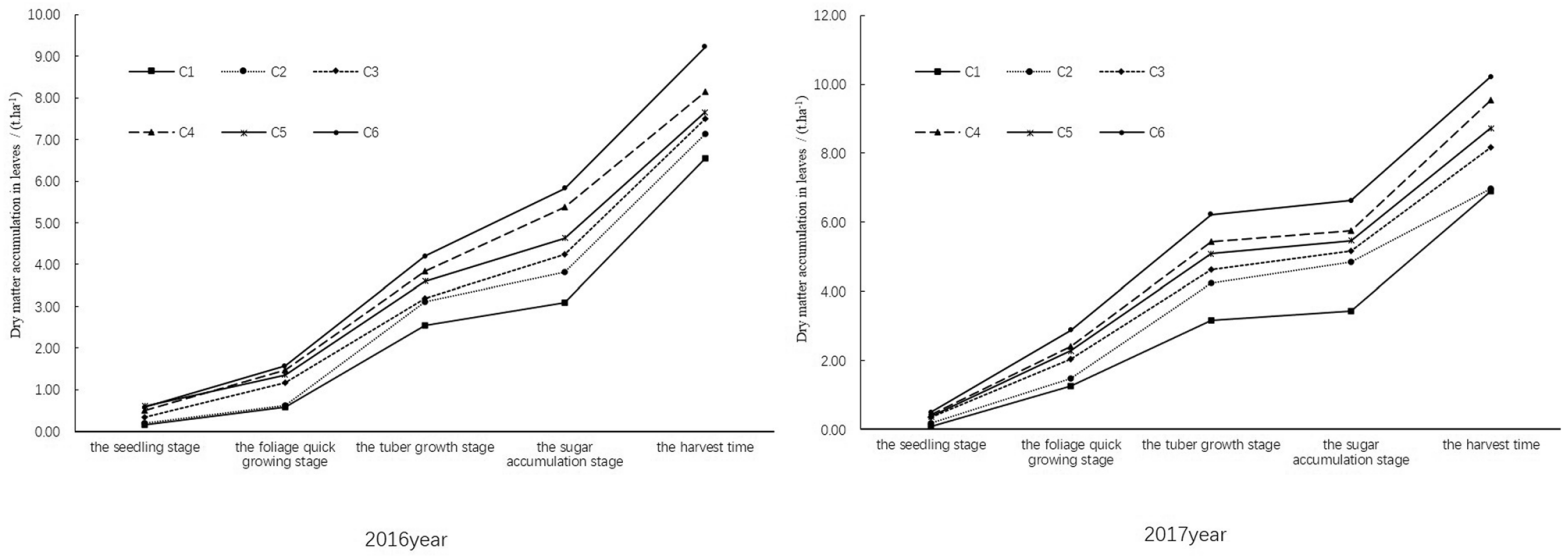
Effect of limited irrigation on dry matter accumulation in the leaves of sugar beet in 2016 and 2017.

Dry matter accumulation in leaves increased with sugar beet growing (Fig. 4), which grew slowly from seedling to foliage rapid growth stages and then rapidly increased at later stages. The maximum leaf dry matter accumulation was observed in C6, being 9.22 ± 0.39 t ha^−1^ and 10.22 ± 0.46 t ha^−1^ in 2016 and 2017, respectively. For the three treatments with nitrogen application (C2, C4 and C6), the leaf dry matter accumulation in C6 was 11.61% and 6.75% more than in C4 and C2 respectively in 2016, and that was 6.75% and 31.70% more than in C4 and C2 respectively in 2017. Under the same irrigation conditions, the nitrogen application in C6 increased dry matter accumulation in leaves by 16.92% and 14.68% in 2016 and 2017 respectively when compared with C5, the nitrogen supply in C4 improved dry matter accumulation in leaves by 7.98% in 2016 and by 14.27% in 2017, and C2 achieved 8.27% and 1.15% more leaf dry matter accumulation than C1 in 2016 and in 2017 respectively. These results showed that both nitrogen application and irrigation increased the dry matter accumulation in leaves of sugar beet.

### Limited irrigation improving nitrogen accumulation in sugar beet

#### Nitrogen accumulation in roots

**Figure 5:**
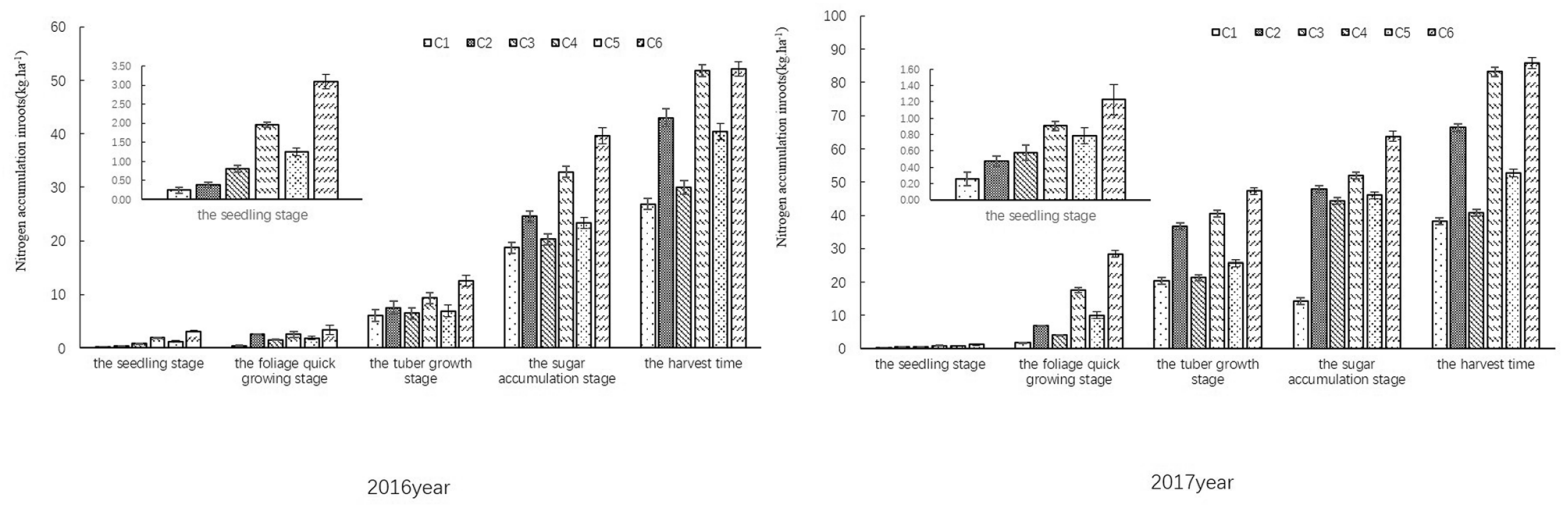
Effect of limited irrigation on nitrogen accumulation rate in the roots of sugar beet in 2016 and 2017.

The tuber nitrogen content of sugar beets showed exponential increase with the growth period progressing (*R*^2^ > 0.9209). As shown in Fig. 5, at every growth stage, the sugar beet plants in treatments with nitrogen (C2, C4 and C6) accumulated more nitrogen in tubers than that in no nitrogen treatments (C1, C3 and C5), and the plants in the treatments with more water supply had more nitrogen in tubers than that in treatments with less water. So, the tuber nitrogen contents of all treatments showed the trend of hole irrigation for seedling + irrigation > hole irrigation for seeding > rain-fed. At seedling stage, the nitrogen content in tubers increased slowly in every treatment, but the index in the treatments using hole irrigation for seedling (C3–C6) was higher than that of rain-fed treatments (C1 and C2). At foliage rapid growth stag, the nitrogen accumulation rate in C2, C4 and C6 was in the order of C6 > C4 > C2, and the nitrogen accumulation rate in C1, C3 and C5 was in the order of C5 > C3 > C1. The nitrogen accumulation rates of C2, C4 and C6 were higher than those of C1, C3 and C5 (*p* < 0.05). At harvest stage, the maximum nitrogen content in tubers was found in C6 in both years, and the value was 52.10 ± 1.32 kg ha^−1^ in 2016 and 85.87 ± 1.72 t ha^−1^ in 2017. We compared C2, C4 and C6 treatments that were applied with the same amount of nitrogen but irrigated by different amount of water. The nitrogen content of tubers in C6 was 17.41% and 0.67% more than in C2 and C4 respectively in 2016; the nitrogen content of tubers in C6 was 22.73% and 3.07% more than in C2 and C4 respectively in 2017. Compared with C1 and C3, the nitrogen content of tubers in C5 was increased by 33.50% and 25.69% respectively in 2016, and it was increased by 22.57% and 27.36% respectively in 2017. We further analyzed the nitrogen content difference between C1 and C2, between C3 and C4, and between C5 and C6. The tuber nitrogen content in C6 was 22.53% and 38.55% more than that in C5 in 2016 and 2017 respectively; the nitrogen content of tubers in C4 was increased by 42.05% in 2016 and by 50.91% in 2017 compared with that in C3; and the nitrogen of tubers in C2 was increased by 37.62% and 42.23% in 2016 and 2017 respectively when compared with C1. These results showed that nitrogen and water supplements both improved the nitrogen accumulation in tubers of sugar beets.

#### Nitrogen accumulation in leaves

**Figure 6:**
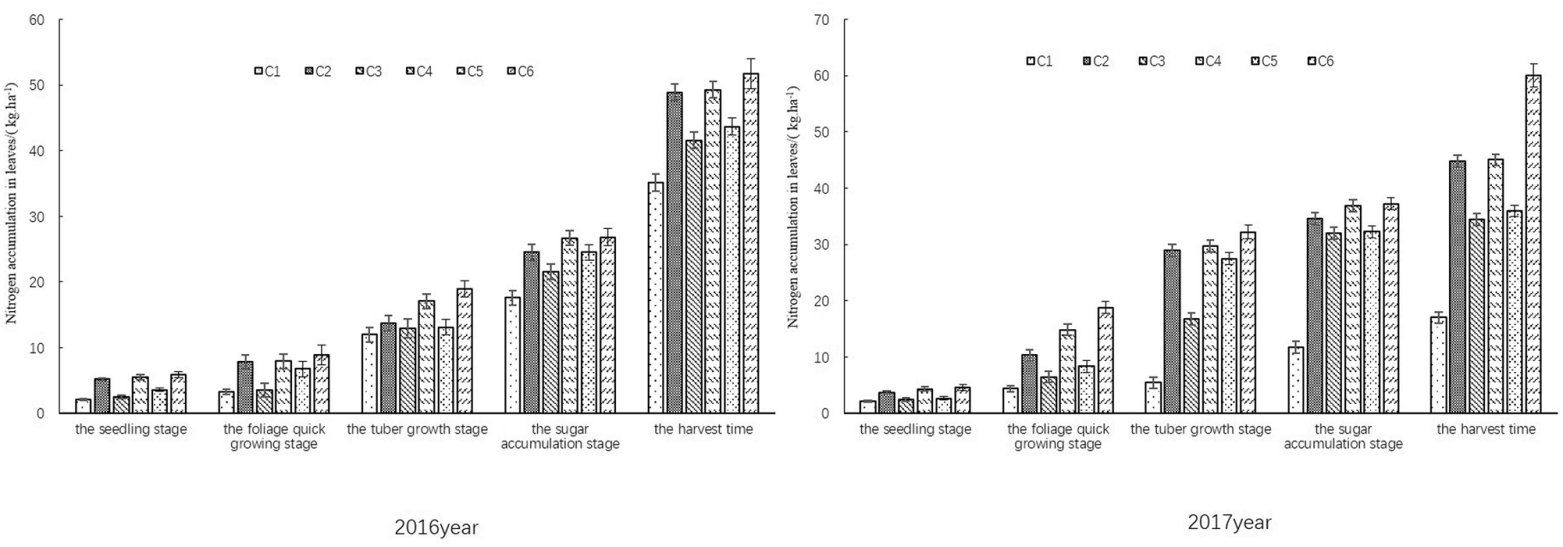
Effect of limited irrigation on nitrogen accumulation rate in the leaves of sugar beet in 2016 and 2017.

With sugar-beet growing, the nitrogen accumulation in leaves of every treatment also increased at an exponential rate (*R*^2^ > 0.9137), and all treatments showed the same trend from seeding to mature stages (Fig. 6). C2, C4 and C6 were applied with the same nitrogen amount but different in irrigation levels. At mature stage, the leaf nitrogen content in C6 was 51.73 ± 2.24 kg ha^−1^ in 2016 and 60.07 ± 2.04 kg ha^−1^ in 2017, which was 4.76% and 5.43% more than that in C4 and C2 respectively in 2016 and was 24.99 and 25.39% more than that in C4 and C2 respectively in 2017. As for the treatments with the same irrigation levels, C6 (120.00 kg ha^−1^ N applied) showed 15.60% and 40.17% more leaf nitrogen content than C5 (no nitrogen applied) in 2016 and 2017 respectively; the leaf nitrogen content of C4 (120.00 kg ha^−1^ N applied) was 15.59% and 23.61% more than of C3 (no nitrogen applied) in 2016 and 2017 respectively; and the 120.00 kg ha^−1^ N applied in C2 increased leaf nitrogen content by 26.57% in 2016 and by 62.05% in 2017 when compared with C1 in which no nitrogen was applied. These results demonstrated that both nitrogen and water supplements improved the nitrogen accumulation in leaves of sugar-beet.

#### Nitrogen use efficiency

**Figure 7:**
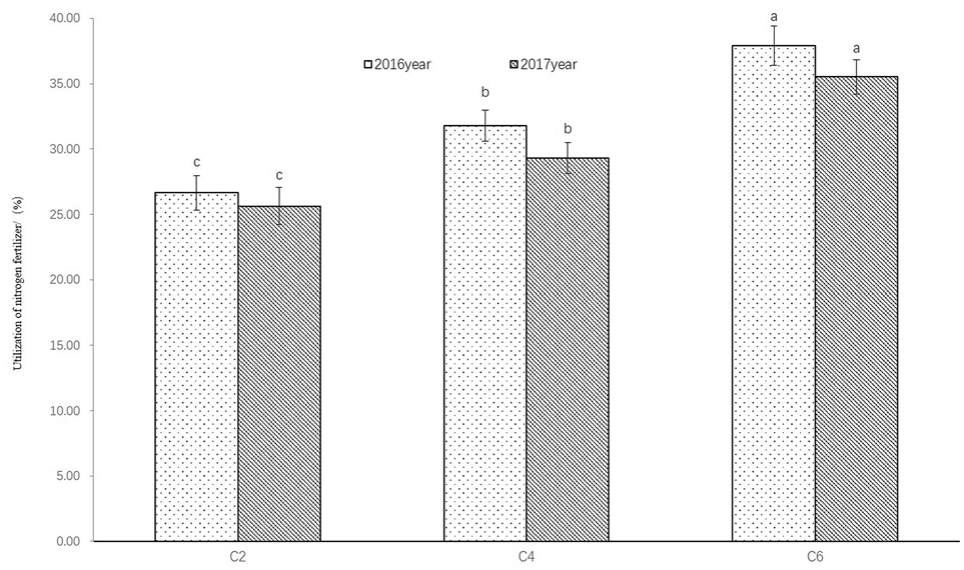
Nitrogen fertilizer utilization under different irrigation in 2016 and 2017. Different lowercase letters in the same column indicate significant differences (*p* < 0.05) among different treatments in the same year.

Nitrogen use efficiency (NUE) was calculated by subtraction method. In both years, C6 and C2 had the highest and lowest NUE among the three treatments applied with nitrogen (C2, C4 and C6) (Fig. 7). The NUE of C6 was 37.93 ± 1.50% in 2016 and 35.51 ± 1.31% in 2017. The NUE differences between the three treatments were significant (*p* < 0.05). The results demonstrated that hole irrigation for seeding and irrigation in growth period improved the NUE in sugar beet. Combined hole irrigation for seeding and irrigation cultivation measures increased NUE by 16.21–17.46% compared with hole irrigation for seeding only (*p* < 0.05), and increased NUE by 12.59–16.17% compared with rain-fed (*p* < 0.05).

### Limited irrigation affecting sugar content and yield of sugar beet

#### Sugar content

Sugar content is the most important economic indicator of sugar beet, which is affected by nitrogen and soil moisture conditions. The result is showed in Table 1, with the progressing of growth period, the sugar content of sugar beet in all treatments increased gradually in the 2 years, and the sugar content in treatments with nitrogen application was higher than that in no nitrogen. At harvest stage, the sugar content differences between nitrogen application treatments and no-nitrogen treatments were significant (*p* < 0.05). Compared with no nitrogen treatment C5, the sugar content in C6 was increased by 3.20% in 2016 and by 6.83% in 2017; compared with C3, the sugar content of C4 was increased by 4.60% in 2016 and by 8.37% in 2017; compared with C1, the sugar content in C2 was increased by 5.69% in 2016 and increased by 7.72% in 2017. Under the same nitrogen level, in 2016 the sugar content of C6 was 2.02% and 12.45% more than that of C4 and C2 respectively, and in 2017, the sugar content of C6 was 5.54% and 7.37% more than that of C4 and C2 respectively. The result showed that the sugar content of sugar beet could be improved by proper irrigation under the same nitrogen conditions.

**Table 1:** Effect of limited irrigation on sugar content of sugar beet at various growth stages.

Treatment	Tuber growth stage (%)	Sugar accumulation stage (%)	Harvest time (%)
**2016**			
C1	10.63 c ± 0.46	12.33 b ± 0.47	13.93d ± 0.31
C2	12.80 b ± 0.40	12.97 b ± 0.50	14.77c ± 0.29
C3	11.60 c ± 0.57	14.20 a ± 0.34	15.77b ± 0.46
C4	13.77 a ± 0.43	14.43 a ± 0.44	16.53a ± 0.22
C5	13.10 ab ± 0.33	14.40 a ± 0.36	16.33ab ± 0.31
C6	13.77 a ± 0.46	14.57 a ± 0.54	16.87a ± 0.39
**2017**			
C1	8.43 c ± 0.26	15.43 c ± 0.15	15.90c ± 0.40
C2	10.80 a ± 0.45	16.20 ab ± 0.44	17.23b ± 0.46
C3	9.63 b ± 0.44	15.83 b ± 0.42	16.10c ± 0.36
C4	10.50 a ± 0.30	16.47 ab ± 0.43	17.57b ± 0.36
C5	9.67 b ± 0.41	16.17 b ± 0.26	17.33b ± 0.35
C6	10.90 a ± 0.35	16.94 a ± 0.35	18.60a ± 0.41

Different lowercase letters in the same column indicate significant differences (*p* < 0.05) among different treatments in the same year.

#### Yield

**Figure 8:**
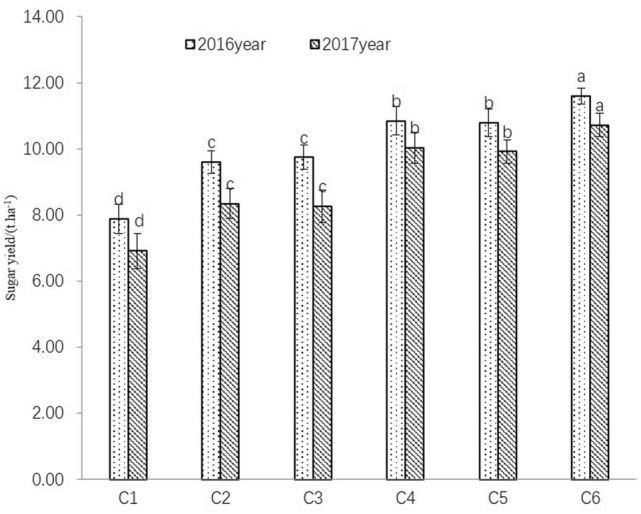
Effect of limited irrigation on the sugar yield of sugar beet in acquisition stage. Different lowercase letters in the same column indicate significant differences (*p* < 0.05) among different treatments in the same year.

Sugar yield is an important index to measure sugar beet production, which can be achieved by multiplying sugar root output and sugar content^23^. In the present study, the treatments supplied with nitrogen and (or) irrigation all got higher sugar yield than rain-fed treatments, and the sugar yield differences between nitrogen treatments and no-nitrogen treatment was significant (*p* < 0.05) (in Fig. 8). In the 2 years, sugar yield of every treatments was in the order of hole irrigation for seeding + irrigation treatment > hole irrigation for seeding treatment > rain-fed treatment. Under the same water conditions, nitrogen application improved sugar yield by 6.94–17.88% in 2016 and by 7.47–17.61% in 2017.

## Discussion

### Limited irrigation and chlorophyll content

Nitrogen affects plant’s photosynthesis and the translocation of assimilates in the aboveground part and roots^42^. Chlorophyll is associated with nitrogen levels and determines the final yield of crops. The increase of nitrogen application can increase the chlorophyll content of sugar beets, and then increase the photosynthetic rate of plants^42,43^. A field experiment conducted by Zhu et al.^44^ showed that the *SPAD* of Chinese cabbage was significantly positively correlated with nitrogen application rate. The study of Wang et al.^45^ showed that the *SPAD* of cotton leaves at different growth stages was significantly positively correlated with total nitrogen content of plants. By studying the photosynthetic characteristics of rice, Yang et al.^46^ concluded that water supplement could significantly increase the chlorophyll content of plants under adequate fertilizer conditions. In our study, the chlorophyll content of leaves was higher in nitrogen treatments than in no-nitrogen treatments, and it was higher in irrigation treatments than in rain-fed treatments. Namely under adequate nitrogen conditions, the water supplement conducted at key growth period of sugar beets helped to improve the final sugar yield, which was consistent with the results based on apple-corn intercropping system^47^ and rice^46^.

### Limited irrigation and the dry matter accumulation, nitrogen content and NUE of sugar beet

Proper irrigation can continuously increase the dry matter and nitrogen accumulation in crops during the whole growth period^48–50^. For example, Wang et al.^51^ found that irrigation significantly affected the nitrogen accumulation and grain yield in wheat. The reasonable combination of nitrogen application and irrigation can improve crops’ NUE, which is of significant for the sustainable development of agriculture^52–54^. In this study, the 2-year experiment demonstrated that the dry matter accumulation and nitrogen amount in sugar beets were positively correlated under various conditions, and they were significantly improved by the cultivation measures of hole irrigation for seeding and (or) irrigation at key growth period. The hole irrigation for seeding and (or) irrigation at key growth period improved NUE too.

### Limited irrigation and the yield of sugar beet

The final yield of sugar beet is determined by tuber yield, sugar content, and plant number per unit area together^18^. Wang et al.^55^ detected that sugar content increased with the increase of nitrogen under the same fertility conditions. Shaw et al.^56^ also concluded that nitrogen fertilizer was conducive to the increase of sugar content and the growth of tubers in sugar beet, and deficient nitrogen supply certainly inhibited the expansion of tubers and then reduced the final yield. The study of Bagherzadeh et al.^57^ showed that nitrogen applied alone could increase the yield of sugar beet by 29–63% and increase the sugar content by 0.2% on average. Our experiments here concluded that nitrogen greatly increased the sugar content and tuber yield of sugar beet. What’s more, water supply could also significantly increase sugar yield of sugar beets under sufficient nitrogen conditions, which is proved by previous studies^48,54,58^ and our study. In the present study, the water supply at foliage rapid growth stage for C6 increased sugar content and sugar yield by 3.78% and 9.91% respectively, and the combination of hole irrigation for seedling and water supply at foliage rapid growth stage increased sugar content and sugar yield by 6.43% and 19.71% respectively. This is may be because the irrigation at key growth stage of sugar beet help them go through the water sensitive period, absorb more nutrients, is favorable for tuber growth, and then increase the final sugar yield.

## Conclusion

The cultivation measures of hole irrigation for seeding together with irrigation at foliage rapid growth stage promote the growth and development of sugar beet and then increase sugar content and yield, and NUE. The 2-year field experiments proved that under the routine fertilization conditions in northeast of China, hole irrigation 500 mL/plant for seeding combined with irrigation 500 mL/plant at foliage rapid growth stage could obtain good yield in sugar beet. The sugar content and yield could reach to 17.74 ± 1.22%, 11.16 ± 0.62 t ha^−1^ respectively, and NUE reached to 36.72 ± 1.71%.
